# Co-infection with feline retrovirus is related to changes in immunological parameters of cats with sporotrichosis

**DOI:** 10.1371/journal.pone.0207644

**Published:** 2018-11-30

**Authors:** Luisa Helena Monteiro de Miranda, Marina Meli, Fátima Conceição-Silva, Marilisa Novacco, Rodrigo Caldas Menezes, Sandro Antonio Pereira, Sarah Sugiarto, Érica Guerino dos Reis, Isabella Dib Ferreira Gremião, Regina Hofmann-Lehmann

**Affiliations:** 1 Laboratory of Clinical Research on Dermatozoonoses in Domestic Animals, Evandro Chagas National Institute of Infectious Diseases, Oswaldo Cruz Foundation, Rio de Janeiro, Brazil; 2 Clinical Laboratory and Center for Clinical Studies, Vetsuisse Faculty, University of Zurich, Zurich, Switzerland; 3 Laboratory of Immunoparasitology, Oswaldo Cruz Institute, Oswaldo Cruz Foundation, Rio de Janeiro, Brazil; CSIRO, AUSTRALIA

## Abstract

Feline sporotrichosis due to *Sporothrix brasiliensis* is frequently severe and often correlated to zoonotic transmission. Feline Immunodeficiency Virus (FIV) and Feline Leukemia Virus (FeLV) cause immunodeficiency in cats; no association has been identified with critical cases of sporotrichosis. Moreover, the cytokine profile in *Sporothrix*-infected cats and a potential impact of retrovirus co-infections on their immunity is unknown. This study assessed immunological parameters in cats with sporotrichosis with and without FIV or FeLV co-infection. FeLV infection was detected by antigen ELISA and by provirus PCR. FIV infection was investigated through ELISA and Western blot. Cytokine transcription (IFN-γ, IL-4, IL-5, IL-6, IL-10, IL-12, TNF-α) was quantified using RT-qPCR and lymphocyte subpopulations (CD4, CD8, CD5 and CD21) were assessed by flow cytometry. Thirty cats with sporotrichosis were recruited to the study, including three FIV-positive and five FeLV-positive (progressive infection) cats. One cat with regressive FeLV infection was excluded from statistics. In comparison to retrovirus-negative cats, FIV-positive cats and FeLV-positive cats had higher IL-10 levels, FeLV-positive cats had lower IL-4 levels and FIV-positive cats had lower IL-12 levels and a lower CD4+/CD8+ ratio. Remarkably, all cats with poor general condition were FeLV (progressive infection) or FIV-positive, but the retrovirus status was not associated with the sporotrichosis treatment length or outcome. The immunological changes and the more severe clinical presentation observed in cats with retrovirus co-infections encourage future prospective studies that address the impact of these changes on prognostic determinants of feline sporotrichosis and the development of new therapy strategies that control disease spread.

## Introduction

Sporotrichosis is a mycosis affecting humans and animals globally, caused by different members of the *Sporothrix schenckii* species complex, including *S*. *brasiliensis*, *S*. *schenckii sensu stricto* (*s*. *str*.), *S*. *globosa*, and *S*. *luriei* [1]. Zoonotic transmission generally occurs through bites or scratches from infected cats [2–3]. Increasing numbers of cases, including in immunocompromised humans, has led to increased interest in this emerging health problem [4–5].

Since 1998, an epidemic outbreak of sporotrichosis in Rio de Janeiro State, Brazil, with increasing number of cases in humans, dogs and cats [6–7] has been characterized by a high rate of transmission from infected cats to humans [8]. The metropolitan region of Rio de Janeiro is now a hyperendemic area for cat-associated sporotrichosis [9] that persists as a neglected zoonosis in this region [3].

Clinically, lesions of feline sporotrichosis are characterized by subcutaneous nodules which ulcerate and are frequently widely disseminated. The outcome of infection in cats ranges from subclinical disease, solitary lesions, to fatal disseminated disease [3, 10–11].

Human sporotrichosis is generally characterized by localized skin lesions or lymphocutaneous lesions, although disseminated disease does occur, frequently associated with conditions of immunosuppression, such as HIV co-infection [5,8]. Feline disseminated sporotrichosis is frequently described in apparently immunocompetent animals [3,10,12].

The most prevalent etiological agent and the primary pathogen of feline sporotrichosis in Brazil is *S*. *brasiliensis* [13]. *Sporothrix brasiliensis* has high virulence and is generally associated with disseminated cutaneous disease in humans. Patients infected with *S*. *schenckii s*. *str*. present with less severe sporotrichosis that can be highly localized [14]. It has been demonstrated that the genotype of *Sporothrix* from cats is identical to *S*. *brasiliensis* from human sources of the endemic area of Rio de Janeiro [13], therefore, the occurrence of cats with different clinical forms of sporotrichosis in this area suggests that other factors may be involved in the disease presentation of feline sporotrichosis.

Feline Immunodeficiency Virus (FIV) and Feline Leukemia Virus (FeLV) are associated with decreased counts of CD4+ T-cells in cats, which may lead to immunodeficiency and to the development of secondary infections [15–19]. The diagnosis of FIV infection in cats is frequently based on the ELISA detection of antibodies against viral proteins such as p24 followed by Western-blot confirmation of inconclusive results [17].

FeLV infection is most commonly identified through ELISA detection of p27 antigen, a marker of infection [20,21]. The development of quantitative real time PCR for the detection of FeLV provirus is crucial for the recognition of FeLV exposure. FeLV provirus-positive/antigen-negative cats may be either at a very early stage of FeLV infection, when antigen is not yet detectable but provirus is, or they may have developed a protective immunity against FeLV and undergone regressive FeLV infection [22]. Cats that are antigen and provirus positive at the time of investigation are generally viremic and may subsequently undergo either regressive or progressive infection. The latter is characterized by persistent antigenemia. Only by repeated determination of antigenemia can progressive and regressive infection outcomes be distinguished [23].

Severe forms of sporotrichosis in cats has not so far been associated with retrovirus co-infection [10–12,24]. However, in previous studies, the cats with sporotrichosis tested for retrovirus co-infection were mainly from Rio de Janeiro, where available resources do not allow confirmation of infection by Western blot or PCR. Assessment of the immune status, as characterized by cytokine expression profiles, has also not been performed in previous cohorts of feline sporotrichosis. The aim of this study was to assess whether the co-infection with FIV or FeLV is related to changes in immunological parameters among cats with sporotrichosis.

## Material and methods

### Sampling

Blood samples were obtained from cats seen at the Laboratory of Clinical Research on Dermatozoonoses of Domestic Animals (LAPCLIN/DERMZOO), Evandro Chagas National Institute of Infectious Diseases (INI), Oswaldo Cruz Foundation (Fiocruz), during the period between April and August, 2013. The opportunistic sampling comprised cats with sporotrichosis, showing skin lesions, with no previous antifungal or corticosteroid therapy. Diagnosis of sporotrichosis was confirmed by isolation and identification of *Sporothrix spp*.

The cats included in the study were divided into three groups according to the distribution of skin lesions: L1, L2 and L3 [10]. Briefly, group L1 included cats with lesions in one location; group L2 included cats with lesions in two non-contiguous locations; and group L3 included cats with lesions in three or more non-contiguous locations. The general condition of the animals was classified as good (absence of extracutaneous signs), fair (presence of mild dyspnea, conjunctivitis, weight loss, dehydration, hypocolored mucosa and prostration), or poor (worsening of the signs described in the fair group), as previously described [24].

Blood samples were obtained through jugular venipuncture and divided into three tubes as follows: 2 mL of blood were transferred to a tube containing EDTA (ethylenediaminetetraacetic acid) for qPCR and RT-qPCR (detection of FeLV provirus and cytokine transcription studies, respectively), 3 mL were transferred to a tube containing heparin (BD Vacutainer, Franklin Lakes, NJ, USA) for peripheral blood mononuclear cells (PBMC) isolation and flow cytometry, and 1 mL was transferred to a tube without anticoagulants (serum for ELISA and Western blot) for the detection of FIV antibodies and ELISA for the detection FeLV p27 antigens. The heparinized blood samples, PBMC samples and serum samples were stored at -80°C, liquid Nitrogen and at -20°C, respectively, until analysis.

The following analyses were performed at the Clinical Laboratory of the Vetsuisse Faculty, University of Zürich, Switzerland. All procedures performed on the animals, as well as the use and storage of their biological samples, were approved by the Animal Use Ethics Committee, FIOCRUZ, under the license number LW-25/14. The owners of the cats included in the study provided a signed informed consent prior to sample collection.

The cats were followed up on an outpatient basis at the LAPCLIN/DERMZOO. The follow-up data regarding the treatment protocol, clinical outcome (cure or failure) and time to cure were obtained from medical records.

After initial sampling and diagnosis, all cats received antifungal therapy with oral itraconazole (ITZ) 100 mg/cat/day or oral ITZ 100 mg/cat/day combined to potassium iodide (KI) capsules 2.5 to 20 mg/Kg/day [11].

The criteria for clinical cure or therapeutic failure were previously described by Reis et al. [11]. Cats that presented with healed skin/mucosal lesions and remission of clinical signs were considered clinically cured. Therapeutic failure was considered the outcome when no clinical improvement was observed for two consecutive appointments; clinical recurrence occurred at any time, or up to 40 weeks of treatment was provided with no clinical cure.

#### Serologic assays for the determination of Feline Leukemia Virus p27 antigenemia and the presence of anti-Feline Immunodeficiency Virus antibodies

To detect FeLV viremia, the presence of FeLV p27 antigen in the plasma was determined using a sandwich ELISA, as previously described [20]. Results were represented as percentages of a defined positive control (culture supernatant of FL-74 feline lymphoblastoid cell line permanently expressing FeLV), which was considered 100%. Samples determined to be > 5% of the positive control signal were considered positive [21].

The serum was tested by ELISA for FIV antibodies using recombinant FIV-Z2 transmembrane glycoprotein (TM- ELISA) as described elsewhere [25]. Samples that reached an OD value > 50% of the positive control were considered positive.

Presence of antibodies to FIV was confirmed by the Western blot (reference standard method) technique as described previously [26]. The presence of both the p24 band and p15 band was interpreted as a positive test result.

#### TaqMan real-time qPCR assay for the detection of Feline Leukemia Virus provirus

DNA was purified from EDTA blood samples using the MagNa Pure LC TNA isolation kit (Roche Diagnostics, Rotkreuz, Switzerland). FeLV proviral DNA was tested by TaqMan real-time qPCR (reference standard method) as previously described [27].

#### Assessment of cytokine profiles

Within one hour after collection, 2 aliquots of 100 μL of whole blood collected in EDTA tubes were admixed with 300 μL mRNA lysis buffer each (mRNA Isolation HS Kit, Roche Diagnostics), respectively and stored at -80° C until subsequent analysis.

The mRNA was purified with mRNA Isolation Kit I and the Magna Pure LC Instrument (Roche Diagnostics) according to the manufacturer's recommendations. For all extractions performed, 100 μL of phosphate Buffered Saline (PBS) was used as negative control, in order to monitor cross-contaminations.

The mRNA was eluted in 25 μL of elution buffer and the first-strand cDNA was synthesized using the High Capacity cDNA Reverse Transcription Kit (Applied Biosystems) according to the manufacturer's instructions. For each sample of mRNA, the cDNA was synthetized in duplicate and pooled.

The TaqMan real-time RT-qPCR assay was used for relative quantification of the following cytokines: interferon-γ (IFN-γ), interleukin (IL) 4, 5, 6, 10 and 12 and tumor necrosis factor-α (TNF-α) as previously described [28–30]. The levels of transcription of the V-abl Abelson murine leukemia viral oncogene (ABL) and zeta polypeptide (YWHAZ)—previously referred as best-performing housekeeping genes in feline blood due to their stable transcription [31]—were employed for normalization. Briefly, to assess the transcription levels of cytokines and reference genes in the samples from this study, a standard curve was acquired using a serial 10-fold dilution of synthetic standards. Afterwards, the transcription levels of the cytokines were normalized to the geometric means of the housekeeping genes, as described elsewhere [32].

#### Assessment of lymphocyte subsets by flow cytometry

The isolation of feline PBMC was carried out using the commercial available density gradient ficoll-hystopaque–density of 1077 g/mL (Histopaque—Sigma-Aldrich, Buchs, Switzerland) as reported previously [24], up to 12 hours after blood collection. The pellets were resuspended in freezing medium with 10% DMSO (Sigma-Aldrich), 50% Fetal Calf Serum (FCS, Hyclone–USA), and 40% RPMI 1640 (Roswell Park Memorial Institute—Sigma–Aldrich) medium and stored in liquid nitrogen.

For the flow cytometry analysis, the PBMC were quickly thawed at 37°C and then resuspended in 10 mL of complete medium (1x RPMI 1640 medium—Sigma-Aldrich, Zug, Switzerland), containing 10% FCS 100 M/mL Glutamine and 1% v/v antibiotic/antimycotic (Ab/Am) (Gibco Life Technologies, Zug, Switzerland), previously warmed to 37° C. The solution was centrifuged (560 x *g*, 10 minutes), the cells were washed once in 10 mL of 1x HBSS (Hank's Balanced Salt Solution, Sigma-Aldrich) and the pellet was resuspended in 1 ml of HBSS. Three aliquots of PBMC were incubated for 30 minutes, at 4°C, with 5 μl of one of the antibody/combination of antibodies, previously diluted in HBSS: 1 –mouse anti-feline CD4-RPE (1:50, Vpg34, ABDserotec, Düsseldorf, Germany); 2 –mouse anti-feline CD8-FITC (1:50, fCD8, Southern Biotech, Allschwil, Switzerland); 3 –mouse anti-feline CD5-FITC (1:100, f43, Southern Biotech) and mouse anti-canine CD21-RPE (1:5, CA2.1D6, ABD Serotec, cross-reacting with feline CD21). Unlabeled samples were employed as negative controls for non-specific binding.

After incubation, the cells were washed twice by centrifugation with HBSS and resuspended in 200 μL of HBSS for acquisition on the flow cytometer. The cytofluorimetric analysis was performed using the Guava EasyCyte 8HT flow cytometer (Millipore, Darmstadt, Germany) using the guavaSoft 2.5 software for data analysis. The population of lymphocytes was defined on the basis of their size and granularity (forward and side scatter) and a gate was set for further analysis. Data were collected from 10,000 cells and are expressed as percentages of positive cells inside the gate.

#### Statistics

Data were stored in and analyzed by the Statistical Package for Social Sciences (SPSS), version 16.0. The following categorical variables were used to group the cats for further analyses: co-infection by FIV or FeLV (positive or negative), general condition (good, fair and poor) and distributions of lesions (L1, L2 and L3). With regards to the co-infection with FeLV, only progressively FeLV-infected cats were included in the statistical analyses and compared to cats without retrovirus infection; cats with regressive FeLV infection were excluded from these analyses. Concerning FIV, cats were considered FIV-positive when they were confirmed to be positive by Western blot. The Pearson’s chi-square test for independence was used to determine a significant association between the categorical variables. Fisher's exact test (pF) was applied to the comparison of variables with only two categories. The Mann-Whitney U-test (p_MWU_) and the Kruskall-Wallis (p_KW_) tests were performed to test for statistical differences in cytokine transcription and percentage of each lymphocyte subset and the treatment length between two groups or among many groups, respectively. P-values below or equal to 0.05 were considered significant.

## Results

### Animals

Thirty cats with sporotrichosis were included in the study. Six cats (20.0%) were included in group L1, ten (33.3%) in group L2 and fourteen (46.7%) in group L3. Twenty-one cats were male (70.0%). Twenty-four (80.0%) were in good general condition, three (10.0%) in fair general condition and three (10.0%) in poor general condition. Fourteen cats (46.7%) were under 2 years old, (<2 years), six (20.0%) were between 2 and five years old (≥ 2 ≤5 years) and seven (23.3%) were more than five years old (>5 years). In three (10.0%) adult cats, age class was unknown.

### Detection of co-infection with feline retroviruses

Among the thirty cats with sporotrichosis included in this study, three were positive for FIV antibodies and six were positive for FeLV provirus (S1 Table); none of the cats was double-positive for FeLV and FIV. All cases that tested positive for FIV antibodies by ELISA tested also positive by FIV Western-blot (detecting also antibodies to FIV); moreover, all cats testing positive for FeLV antigen by ELISA tested also positive by FeLV real-time qPCR assay (detection of FeLV provirus). The use of ELISA alone resulted in one false negative case of FIV; the cat was FIV-ELISA negative but FIV Western blot positive and was regarded as FIV-infected. One FeLV-exposed cat was provirus positive without detectable antigenemia. This cat was not considered in the statistical analyses concerning retrovirus infections since it was categorized as having regressive FeLV infection.

A poor general condition was observed in two out of three FIV positive cats and it was associated to FIV infection when compared to retrovirus-negative cats (p_F_ < 0.01). In cats with progressive FeLV infection, a poor general condition was observed in one out of five cats. Among cats that tested negative for both retroviruses (n = 21), 95.2% were in a good general condition, 4.8% were in a fair general condition and none of them presented a poor general condition. Thus, all the cats that presented with poor general condition were co-infected with either FeLV (progressive infection) or FIV while none of the retrovirus-negative cats presented poor general condition (p_F_ = 0.01).

### Assessment of cytokine profile

The assessment of cytokine profile by means of quantitative RT-PCR was performed in all 30 animals. When comparing the 21 retrovirus-negative cats with sporotrichosis, no significant differences were found among cats in groups L1, L2 and L3 in cytokine transcription in any of the tested cytokines (S2 Table). It was not possible to compare the cytokine levels among cats with no retrovirus co-infection according to their general condition due to the small number (n = 1) of animals in fair general condition.

**Fig 1:**
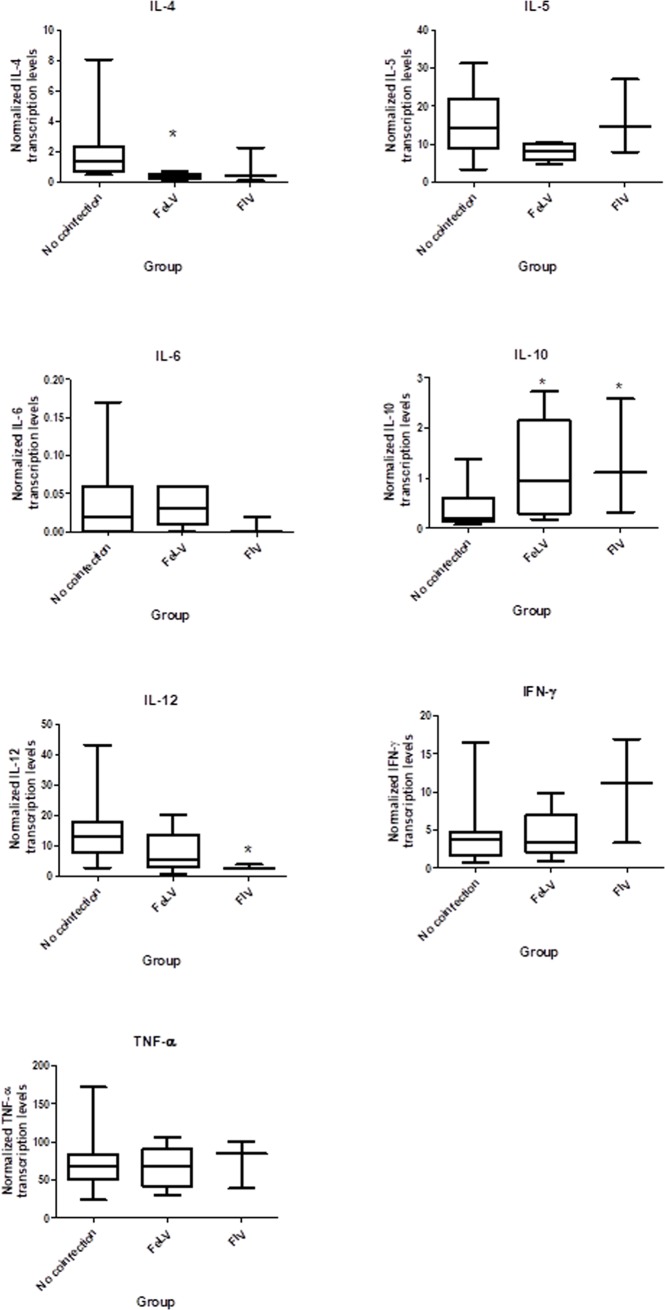
Relative cytokine transcription levels by means of RT-qPCR in peripheral blood of cats with sporotrichosis co-infected or not with Feline Leukemia Virus (FeLV; progressive infection) or Feline Immunodeficiency Virus (FIV). (A) IL-4, (B) IL-5, (C) IL-6, (D) IL-10, (E) IL-12, (F) IFN-γ and (G) TNF-α. The cytokine transcription levels were normalized to the geometric means of the housekeeping genes (y-axes). IL-4 was significantly lower (P_MWU_ = 0.02) in cats with progressive FeLV infection, while IL-12 was significantly lower (P_MWU_ = 0.01) in FIV-positive cats, in comparison to retrovirus-negative cats. IL-10 was significantly higher in both cats with progressive FeLV infection (P_MWU_ = 0.04) and in FIV-positive cats (P_MWU_ = 0.05), in comparison to retrovirus-negative cats. *P_MWU_≤0.05.

When analyzing the cytokine levels according to the retrovirus status, significantly higher IL-10 transcription levels were found in FIV-positive cats and in cats with progressive FeLV infection compared to retrovirus-negative cats (Fig 1). Moreover, cats with progressive FeLV infection also had significantly lower IL-4 levels and FIV-infected cats had significantly lower IL-12 (Fig 1) when compared to retrovirus-negative cats. No significant differences were found in IL-6, IFN-γ and TNF-α transcription levels.

### Evaluation of lymphocytes subsets by flow cytometry

This analysis was done in 29 out of the 30 cats in the study, since live cell recovery after thawing was too low to perform the assay in one FeLV-positive cat. There were no significant differences in the lymphocyte subsets among the cats in groups L1, L2 and L3 (S3 Table).

**Fig 2:**
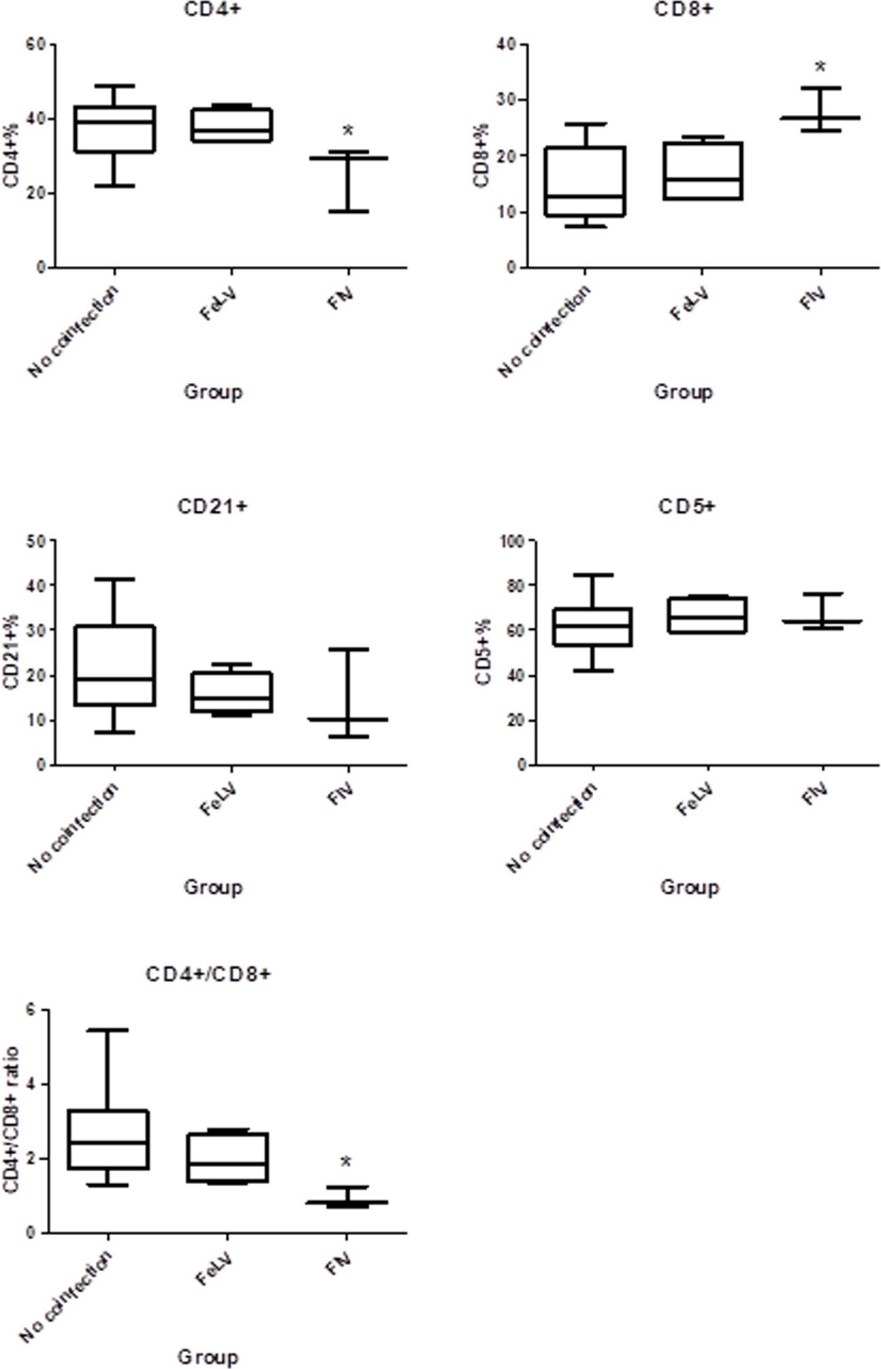
Evaluation of median percentages of lymphocyte subsets by means of flow cytometry in peripheral blood of cats with sporotrichosis co-infected or not with Feline Leukemia Virus (FeLV; progressive infection) or Feline Immunodeficiency Virus (FIV). (A) CD4+ cells, (B) CD8% cells, (C) CD21+ cells, (D) CD5+ cells and (E) CD4+/CD8+ ratio. FIV-positive cats presented lower percentages of CD4+ cells (P_MWU_ = 0.04), higher percentages of CD8+ cells (P_MWU_<0.01) and lower CD4+/CD8+ ratio (P_MWU_ = 0.006), in comparison to retrovirus-negative cats. *P_MWU_≤0.05.

When considering retroviral infections, FIV-positive cats with sporotrichosis had significantly lower CD4+ and higher CD8+ cells than retrovirus-negative cats with sporotrichosis (Fig 2). The inversion of CD4:CD8 ratio was present in two out of three FIV-positive cats, but was not observed in cats with progressive FeLV infection or in retrovirus-negative cats. FIV-positive cats had significantly lower CD4:CD8 ratio compared to retrovirus-negative cats (P_MWU_ = 0.006). No significant differences were observed between FIV-positive and retrovirus-negative cats in CD5 (feline T cells) and CD21 (B cells). Additionally, there was no significant difference in any of the tested lymphocytes subsets when cats with progressive FeLV infection were compared to retrovirus-negative cats.

### Clinical follow-up

Based on the data collected from the medical records, 24 cats were followed up until treatment outcome. Four retrovirus-negative cats and one cat with progressive FeLV infection were lost to follow up.

Among the remaining retrovirus-negative cats (n = 17), clinical cure was observed in sixteen and therapeutic failure in one cat. Among the remaining cats with progressive FeLV infection (n = 4), three were treated with ITZ+KI and all of them were cured. All FIV-positive cats (n = 3) were treated with ITZ alone and two of them were cured. The cure rates were not related to the treatment protocol or to the retrovirus status. The S4 Table shows therapeutic aspects of the cats from this study according to their retrovirus status.

The treatment length was not statistically different between the groups, regardless of therapeutic protocol or retrovirus status. Among cats that were clinically cured, the overall median treatment length was 12 weeks: 12 weeks in retrovirus-negative cats, 9.5 weeks in cats with progressive FeLV infection and 20.5 weeks in FIV-positive cats. The median treatment length in retrovirus-negative cats was higher in the therapeutic protocol using ITZ alone (median = 12 weeks) when compared to ITZ + KI (median = 9 weeks). In cats with progressive FeLV infection, the treatment length was also higher in cats treated with ITZ alone (median = 13 weeks) in comparison to those treated with ITZ+KI (median = 9 weeks).

## Discussion

In this study, 30 cats with sporotrichosis were investigated for feline retrovirus co-infection and immunological aspects based on cytokine expression profiles of peripheral blood mononuclear cells. To the best our knowledge, this is the first study to assess cytokine transcription in the blood of cats with sporotrichosis.

Previous studies have not established a correlation between severe sporotrichosis and feline retrovirus co-infections [10,12,24] likely explained by the use of clinical and therapeutic aspects in case definitions. Recently, Miranda et al. [24] described the leukocyte profile in blood of cats with sporotrichosis, but because there were no FIV co-infected cats and the number of FeLV co-infected cats was limited, no statistical correlation with leukocyte profile could be established. In the present study, there was also a low incidence of FeLV and FIV infected cats; this may have reduced the statistical power for detecting significant differences potentially caused by retrovirus infections.

The low incidence of retrovirus infections in most study populations of cats and the lack of confirmation of positive test results in other studies using gold standard methods have led to an increase in the likelihood of false positive cases identified in previous reports (low positive predictive value of the screening results).

In the current study, for diagnosing FIV status in cats, the Western blot technique, which is considered the gold standard method for detection of FIV antibodies in areas without vaccination [17], was applied. The occurrence of false negative cases with the use of ELISA has been previously reported in Calzolari *et al*. [25], when four out of 194 cats tested negative for the FIV TM ELISA and positive in the Western blot. As previously observed, that lack of reactivity with the recombinant TM of some FIV infected domestic cats may indeed be due to infection with a FIV distinct from that the TM antigen utilized in the ELISA [25].

Real-time PCR was employed to detected and quantify FeLV provirus in addition to FeLV p27 antigen detection by ELISA. PCR is a more sensitive measure for FeLV exposure than antigen detection. FeLV provirus-positive/antigen-negative cats have been described as having regressive FeLV infection [22]. In the current study, only one cat had a regressive FeLV-infection. Because of the small number (n = 1) of FeLV regressive infection cases, this cat was not included in the statistical analysis as a separate group. Moreover, it was also not included with the cats with progressive FeLV infection cats, since the cat was not expected to present major immunological changes at this stage. However, since this cat is latently infected, a subsequent reactivation of the viremia cannot be ruled out, especially in cases of comorbidities, immunosuppression or chronic stress [18]. Cats with regressive FeLV infection should be further monitored for reappearance of FeLV antigen; if they become positive again, they need to be considered as potential sources of FeLV infection and will most probably show immunological changes [18].

Studies regarding immune response in human sporotrichosis are scarce; however the correlation between severe sporotrichosis and co-infection with HIV has been established [33–35]. The clinical and pathological presentation of sporotrichosis in HIV-positive patients [34] is often comparable to that seen in cats with sporotrichosis, whether co-infected with retrovirus or not [3,36]. This study shows a significant association between the poor general condition in cats with sporotrichosis and co-infection with FIV or with feline retroviruses in general, suggesting that retrovirus co-infections may influence the clinical presentation in cats with sporotrichosis.

The reduced transcription levels of IL-12 in FIV co-infected cats when compared to retrovirus-negative cats in this study suggested alteration and perhaps compromise of the Th1 cellular immune response in these cats. IL-10 was significantly increased in cats with sporotrichosis co-infected with either FIV or FeLV, leading to a potential immunosuppressive condition, since this cytokine is widely involved with immunoregulatory effects [37]. IL-10 is also reported to suppress cellular immune response by inhibiting cytokine production by Th1 cells and activated macrophages [38–39].

FIV-positive cats are susceptible to some intracellular pathogens, including *Toxoplasma gondii* and *Listeria monocytogenes*, even in early stages of the retrovirus infection, when clinical signs are not yet observed, due to increasing levels of IL-10 and decreased levels of IL-12 [40–42], such as described in FIV-infected cats in the present study. The balance between the anti-inflammatory IL-10 and pro-inflammatory cytokines, such as IL-12, seems to be a key factor for an adequate inflammation in fungal infection, since IL-10 might be required for limiting high levels of inflammation and the consequent host damage [43]. In experimental murine sporotrichosis, IL-10 is involved with impairment of fungicidal activity of macrophage, as well as with unresponsiveness of T-cells [44]. In humans, high levels of IL-10 production in response to some viral infections plays a role in viral persistence, by suppressing immune functions [45–47]. The high levels of IL-10 in FIV-positive cats in this study may be related to a high percentage of regulatory T (Treg) cells, since these cells can be directly activated by FIV and are normally increased in infected animals [48]. Regulatory T cells were not measured in this study.

Ferreira et al. [49] have recently shown that Th17 immunity is required for the optimal fungal clearance in the murine model of systemic sporotrichosis. Although the role of Th17 arm is still unknown in feline sporotrichosis, the levels of IL-6, a cytokine which induces the development of Th17 cells, did not differ among the cats from this study. This suggests that the retrovirus co-infection and the upregulation of IL-10, with a potential polarization to a Treg cell profile, did not impair Th17 immunity. This is relevant because despite having opposite functions, Th17 and Treg cells develop from CD4+ cell precursors through the same pathway, but depending upon the presence of different cytokines [50]. Despite the very low levels of IL-6 detected in the cats from this study, it is not possible to suggest that Th17 profile is depressed in favor of Tregs increase in retrovirus co-infection, due to the small number of FIV-positive cats. Another limitation of this study was the absence of a control group of healthy cats, so that it cannot be confirmed whether IL-6 levels are altered in cats with sporotrichosis, co-infected with retrovirus infections or not, in comparison to healthy cats.

Although the first steps of fungus-host interactions are considered determinant for further adaptive response in the murine model [51–54], the scarcity of information on innate immunity in feline sporotrichosis remains a concern. A detrimental role of nitric oxide (NO) in the murine immune response to *S*. *schenckii* was reported and related to T-cell suppression and fungal dissemination, which was attributed to increased apoptosis and significant elevations of IL-10 and TNF-α [44,54]. Interestingly, FIV co-infected cats from this study presented higher levels of IL-10 (P_MWU_ = 0.05). In addition, it has been shown that PD-1, the programmed cell death protein 1, is upregulated in FIV-infected lymphocytes and may correlate with dysfunction or exhaustion of T cells and with immunosuppression in infected cats [55]. It could be speculated that the cytokine changes in cats with sporotrichosis coinfected with FIV may be at some level linked to the large number of yeast commonly found in cutaneous lesions of feline sporotrichosis. However, further work is required to address these issues properly.

The evaluation of lymphocyte subsets by flow cytometry showed a lower percentage of CD4+ cells and a higher percentage of CD8+ cells, as well as a lower CD4:CD8 ratio in FIV-positive cats, when compared to retrovirus-negative cats. This finding is consistent with what has been reported for FIV infection, in which the inversion of CD4:CD8 ratio is commonly detected, along with decreasing CD4+ levels and increasing CD8+ levels [56]. This might be linked to the low concentration of IL-12 in FIV-positive cats described in this study, as well as to their poor general condition.

Cellular immunity was recently referred to as crucial for controlling feline sporotrichosis [24], presumably through Th1-driven mechanisms, with increased percentages of CD4+ cells in cats with good general condition, localized lesions, a well-organized granulomatous inflammatory response and a low fungal burden. In humans, the decrease in CD4+ cell counts also correlated with disseminated sporotrichosis [57]. The poor general condition and the disseminated lesions with high fungal burden in cats with sporotrichosis were related to higher CD8^low^+ cells percentage, which is suggested to be elicited by a Th2 immunity background [58]. In coronavirus infection, cats that did not develop Feline Infectious Peritonitis (FIP), a fatal granulomatous disease, had higher tissue levels of IL-10, which probably prevented excessive macrophage activation and the development of FIP [59]. Although further studies are still necessary to determine the mechanisms driving ineffective granulomas in feline sporotrichosis, it could be considered that, as described in coronavirus-infected cats, the retrovirus co-infection with upregulation of IL-10 is possibly involved with the impairment of granuloma formation and increasing fungal load in lesions of feline sporotrichosis.

Cats with severe sporotrichosis clearly outnumbered retrovirus co-infected cats. Thus, whether cats with severe sporotrichosis and negative for FIV and FeLV are truly free of other comorbidities or immunosuppressive conditions remains unknown. Since most of the areas in which sporotrichosis occurs in Rio de Janeiro have low educational and socioeconomic status and poor health assistance [5,60–61], infectious diseases other than sporotrichosis can spread. In addition, the vaccination and prophylactic deworming status in the population of cats with sporotrichosis have yet to be determined [62].

Certainly, helminth infections typically lead to an increased production of IL-10 and Th2 cytokines, such as IL-4 [63–64], and to the down-regulation of Th1 cytokines [63]. An experimental study in rats with sporotrichosis showed that different profiles of cytokines are driven depending on the coinfection with *Taenia taeniaeformis* [65]. The authors demonstrated that IL-10 was increased in rats co-infected with the worm. These animals showed a less organized inflammatory infiltrate, and a higher fungal burden. As previously speculated for FIV, the *Sporothrix*/helminths co-infection could lead to the imbalance of cytokines, resulting in impaired response to *Sporothrix* and in the lack of control over the fungal infection in cats.

Therefore, other comorbidities should be considered as potential challenges and the presence of immunosuppressive conditions during the course of sporotrichosis in cats could explain the occurrence of relapses, the worsening on clinical condition after the beginning of the treatment and therapeutic failure.

In this study, the immunological changes related to retrovirus co-infection in cats with sporotrichosis were not associated with a difference in the clinical outcome or duration of treatment. Still, the combination of ITZ and KI in the treatment of cats decreased the median treatment length in cats in this study. Even though this reduction was not statistically significant, it is important to state that the combination of these drugs had previously shown promising results in the treatment of feline sporotrichosis [11]. While the effectiveness of KI is not proved to be due to the direct action on fungi [66], the mechanism through which its use is successful remains obscure. Although this issue still needs to be addressed, a potential immunomodulation is suggested [67–69] and could have contributed to the shorter treatment length in cats treated with KI in this study, especially on FeLV co-infection. However, this study had a retrospective design, with retrospective evaluation of medical records. Prospective double blinded controlled studies should be performed to properly assess effectiveness of the different therapeutic regimes.

Despite the efforts towards the better understanding of interactions between *Sporothrix* spp. and feline immune system, much of the progress that has been performed *in vitro* and *in vivo* experimental models is still to be explored in cats. Retrovirus/*Sporothrix* co-infection in cats from this study led to important changes in immunological balance, leaning towards an immunosuppressive profile with higher levels of IL-10, which could prevent the activation of a Th1 response that would be favourable against sporotrichosis. The consequent imbalance in host-pathogen interactions may potentially result in higher availability of *Sporothrix* in skin lesions and increased transmission from cats to humans. Indeed, all cats that had the most severe clinical presentation and poor general condition were co-infected with FeLV or FIV. Therefore, these results encourage the development of prospective longitudinal studies that address the impact of immunological changes in the outcome of cats with sporotrichosis. This would be the key to better understand prognostic determinants of feline sporotrichosis and to developing new therapy strategies to control disease spread.

## Supporting information

S1 Table Evaluation of the co-infection with FIV and FeLV in cats with sporotrichosis by means of different techniques. Three cats were positive for FIV and six for FeLV; none of the cats was double-positive for FeLV and FIV. All positive cases by ELISA tested also positive by the reference standard methods for both retrovirus, whereas the use of ELISA alone resulted in one false negative case for the diagnosis of each retroviruses.
(DOCX)

S2 Table Assessment of cytokines levels by quantitative RT-qPCR in the 21 cats with sporotrichosis, without retrovirus co-infection, and their correlation with the groups L1, L2 and L3. (DOCX)

S3 Table Evaluation of percentages of lymphocytes subsets in peripheral blood of cats with sporotrichosis by flow cytometry and its correlation with the groups L1, L2 e L3. (DOCX)

S4 Table Therapeutic aspects of cats with sporotrichosis according to retrovirus status. (DOCX)
