# Supplementary material for: Co-infection with feline retrovirus is related to changes in immunological parameters of cats with sporotrichosis
Source: PLoS One. 2018 Nov 30;13(11):e0207644. doi: 10.1371/journal.pone.0207644 (PMC6267967; doi:10.1371/journal.pone.0207644)
Supplement: S1 Table — Three cats were positive for FIV and six for FeLV; none of the cats was double-positive for FeLV and FIV. All positive cases by ELISA tested also positive by the reference standard methods for both retrovirus, whereas the use of ELISA alone resulted in one false negative case for the diagnosis of each retroviruses. (DOCX) [file pone.0207644.s001.docx]

**S1 Table.** **Evaluation of the co-infection with FIV and FeLV in cats with sporotrichosis by means of different techniques.** Three cats were positive for FIV and six for FeLV; none of the cats was double-positive for FeLV and FIV. All positive cases by ELISA tested also positive by the reference standard methods for both retrovirus, whereas the use of ELISA alone resulted in one false negative case for the diagnosis of each retroviruses.

| **Sample** | **FeLV** | | **FIV** | |
| --- | --- | --- | --- | --- |
|  | **ELISA** | **PCR** | **ELISA** | **Western-Blot** |
| **1** | Negative | Negative | Negative | **Positive** |
| **2** | Negative | Negative | Negative | Negative |
| **3** | Negative | Negative | Negative | Negative |
| **4** | Negative | Negative | **Positive** | **Positive** |
| **5** | Negative | Negative | Negative | Negative |
| **6** | Negative | Negative | Negative | Negative |
| **7** | **Positive** | **Positive** | Negative | Negative |
| **8** | Negative | Negative | Negative | Negative |
| **9** | Negative | Negative | Negative | Negative |
| **10** | Negative | Negative | Negative | Negative |
| **11** | Negative | Negative | Negative | Negative |
| **12** | Negative | Negative | Negative | Negative |
| **13** | **Positive** | **Positive** | Negative | Negative |
| **14** | Negative | Negative | Negative | Negative |
| **15** | **Positive** | **Positive** | Negative | Negative |
| **16** | Negative | Negative | Negative | Negative |
| **17** | Negative | Negative | Negative | Negative |
| **18** | Negative | Negative | Negative | Negative |
| **19** | **Positive** | **Positive** | Negative | Negative |
| **20** | Negative | Negative | Negative | Negative |
| **21** | Negative | Negative | Negative | Negative |
| **22** | Negative | Negative | Negative | Negative |
| **23** | Negative | Negative | Negative | Negative |
| **24** | Negative | Negative | Negative | Negative |
| **25** | Negative | Negative | **Positive** | **Positive** |
| **26** | Negative | **Positive** | Negative | Negative |
| **27** | Negative | Negative | Negative | Negative |
| **28** | Negative | Negative | Negative | Negative |
| **29** | Negative | Negative | Negative | Negative |
| **30** | **Positive** | **Positive** | Negative | Negative |
