# Supplementary material for: Co-infection with feline retrovirus is related to changes in immunological parameters of cats with sporotrichosis
Source: PLoS One. 2018 Nov 30;13(11):e0207644. doi: 10.1371/journal.pone.0207644 (PMC6267967; doi:10.1371/journal.pone.0207644)
Supplement: S2 Table — (DOCX) [file pone.0207644.s002.docx]

**S2 Table.** **Assessment of cytokines levels by quantitative RT-qPCR in the 21 cats with sporotrichosis, without retrovirus co-infection, and their correlation with the groups L1, L2 and L3.**

|  | **IL-4** | | **IL-5** | | **IL-6** | | **IL-10** | | **IL-12** | | **IFN-γ** | | **TNF-α** | |
| --- | --- | --- | --- | --- | --- | --- | --- | --- | --- | --- | --- | --- | --- | --- |
| **Group** | **Median**  **(Min-Max)** | **n** | **Median**  **(Min-Max)** | **n** | **Median**  **(Min-Max)** | **n** | **Median**  **(Min-Max)** | **n** | **Median**  **(Min-Max)** | **n** | **Median**  **(Min-Max)** | **n** | **Median**  **(Min-Max)** | **n** |
| **L1** | 1.80 | 5 | 16.45 | 5 | 0.05 | 3 | 0.15 | 5 | 15.09 | 5 | 3.94 | 5 | 80.46 | 5 |
|  | (0.53-8.06) |  | (9.21-27.34) |  | (0.00-0.17) |  | (0.07-1.37) |  | (5.90-25.35) |  | (0.79-6.83) |  | (23.64-120.76) |  |
| **L2** | 1.47 | 8 | 9.43 | 8 | 0.06 | 6 | 0.37 | 8 | 12.42 | 8 | 3.01 | 8 | 68.05 | 8 |
|  | (0.60-2.86) |  | (3.64-25.00) |  | (0.02-0.10) |  | (0.09-0.96) |  | (2.92-17.78) |  | (0.87-16.58) |  | (39.28-172.37) |  |
| **L3** | 0.95 | 8 | 15.42 | 8 | 0.04 | 4 | 0.21 | 8 | 11.92 | 8 | 3.28 | 8 | 63.13 | 8 |
|  | (0.51-6.17) |  | (3.44-31.30) |  | (0.00-0.09) |  | (0.07-0.65) |  | (3.53-43.01) |  | (1.47-5.92) |  | (48.75-86.58) |  |
| **P_KW_^1^** | 0.79 | | 0.35 | | 0.38 | | 0.50 | | 0.74 | | 0.98 | | 0.80 | |

L1: cats with lesions in one location; L2: cats with lesions in two non-contiguous locations; L3: cats with lesions in three or more non-contiguous locations.

Note: Min = Minimum, Max = Maximum. ^1^Kruskal Wallis test
