# Supplementary material for: Co-infection with feline retrovirus is related to changes in immunological parameters of cats with sporotrichosis
Source: PLoS One. 2018 Nov 30;13(11):e0207644. doi: 10.1371/journal.pone.0207644 (PMC6267967; doi:10.1371/journal.pone.0207644)
Supplement: S3 Table — (DOCX) [file pone.0207644.s003.docx]

**S3 Table.** **Evaluation of percentages of lymphocytes subsets in peripheral blood of cats with sporotrichosis by flow cytometry and its correlation with the groups L1, L2 e L3.**

|  | **CD4** | | **CD8** | | **CD5** | | **CD21** | |
| --- | --- | --- | --- | --- | --- | --- | --- | --- |
| **Group** | **Median**  **(Min-Max)** | **n** | **Median**  **(Min-Max)** | **N** | **Median**  **(Min-Max)** | **n** | **Median**  **(Min-Max)** | **n** |
| **L1** | 39.20 | 5 | 12.55 | 5 | 62.48 | 5 | 24.17 | 5 |
|  | (24.68-40.39) |  | (8.09-25.73) |  | (50.68-68.75) |  | (12.33-41.40) |  |
| **L2** | 38.85 | 8 | 10.12 | 8 | 57.24 | 8 | 22.99 | 8 |
|  | (21.96-48.70) |  | (7.3-22.15) |  | (41.96-84.92) |  | (7.29-35.02) |  |
| **L3** | 36.82 | 8 | 19.04 | 8 | 63.90 | 8 | 17.47 | 8 |
|  | (29.07-45.48) |  | (9.17-24.28) |  | (51.58-76.45) |  | (11.65-36.72) |  |
| **P_KW_^1^** | 0.89 | | 0.09 | | 0.53 | | 0.76 | |

L1: cats with lesions in one location; L2: cats with lesions in two non-contiguous locations; L3: cats with lesions in three or more non-contiguous locations. Note: Min = Minimum, Max = Maximum. ^1^ Kruskal Wallis test
