# Supplementary material for: Co-infection with feline retrovirus is related to changes in immunological parameters of cats with sporotrichosis
Source: PLoS One. 2018 Nov 30;13(11):e0207644. doi: 10.1371/journal.pone.0207644 (PMC6267967; doi:10.1371/journal.pone.0207644)
Supplement: S4 Table — (DOCX) [file pone.0207644.s004.docx]

|  |  | **Retrovirus Negative cats (N=17)** | **FeLV-positive cats**  **(N=4)** | **FIV-positive cats**  **(N=3)** |
| --- | --- | --- | --- | --- |
| **Treatment protocol** | **ITZ** | 10 | 3 | 3 |
|  | **ITZ+KI** | 7 | 1 | 0 |
| **Clinical outcome** | **Clinical cure** | 16 | 4 | 2 |
|  | **Treatment failure** | 1 | 0 | 1 |
| **Median**  **treatment length (weeks)** | **Total** | 12 | 9.5 | 20.5 |
|  | **ITZ** | 12 | 13 | 20.5 |
|  | **ITZ+KI** | 9 | 9 | - |

**S4 Table. Therapeutic aspects of cats with sporotrichosis according to retrovirus status.**
